# Supplementary material for: Damage to the Testicular Structure of Rats by Acute Oral Exposure of Cadmium
Source: Int J Environ Res Public Health. 2021 Jun 4;18(11):6038. doi: 10.3390/ijerph18116038 (PMC8200047; doi:10.3390/ijerph18116038)
Supplement: Supplementary file 1 [file ijerph-18-06038-s001.zip › ijerph-1158375-SI.pdf]

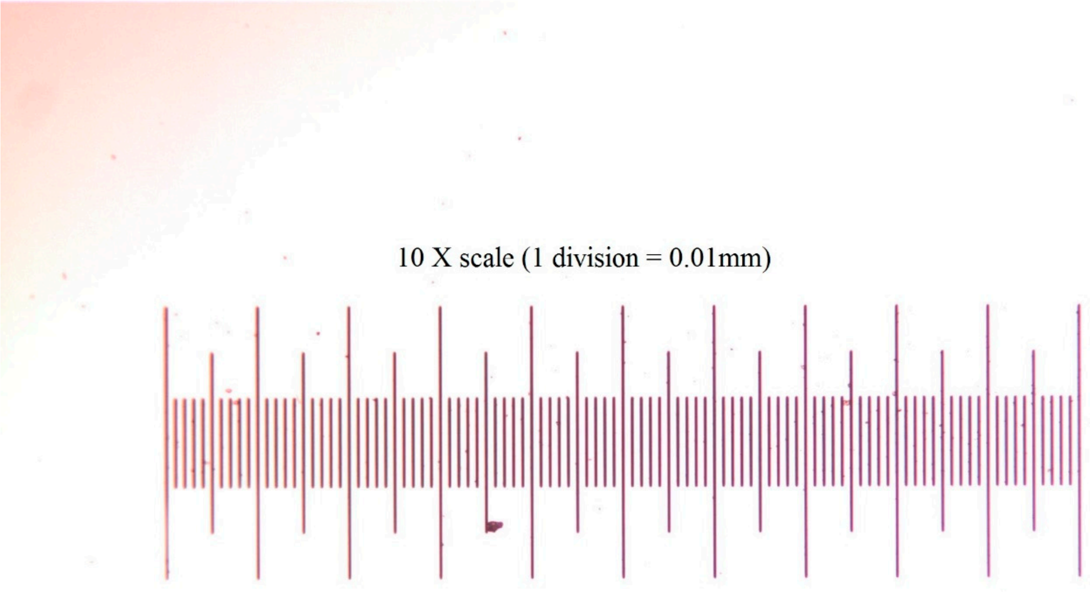

Supplemental figure: 10X scale for histological calculations

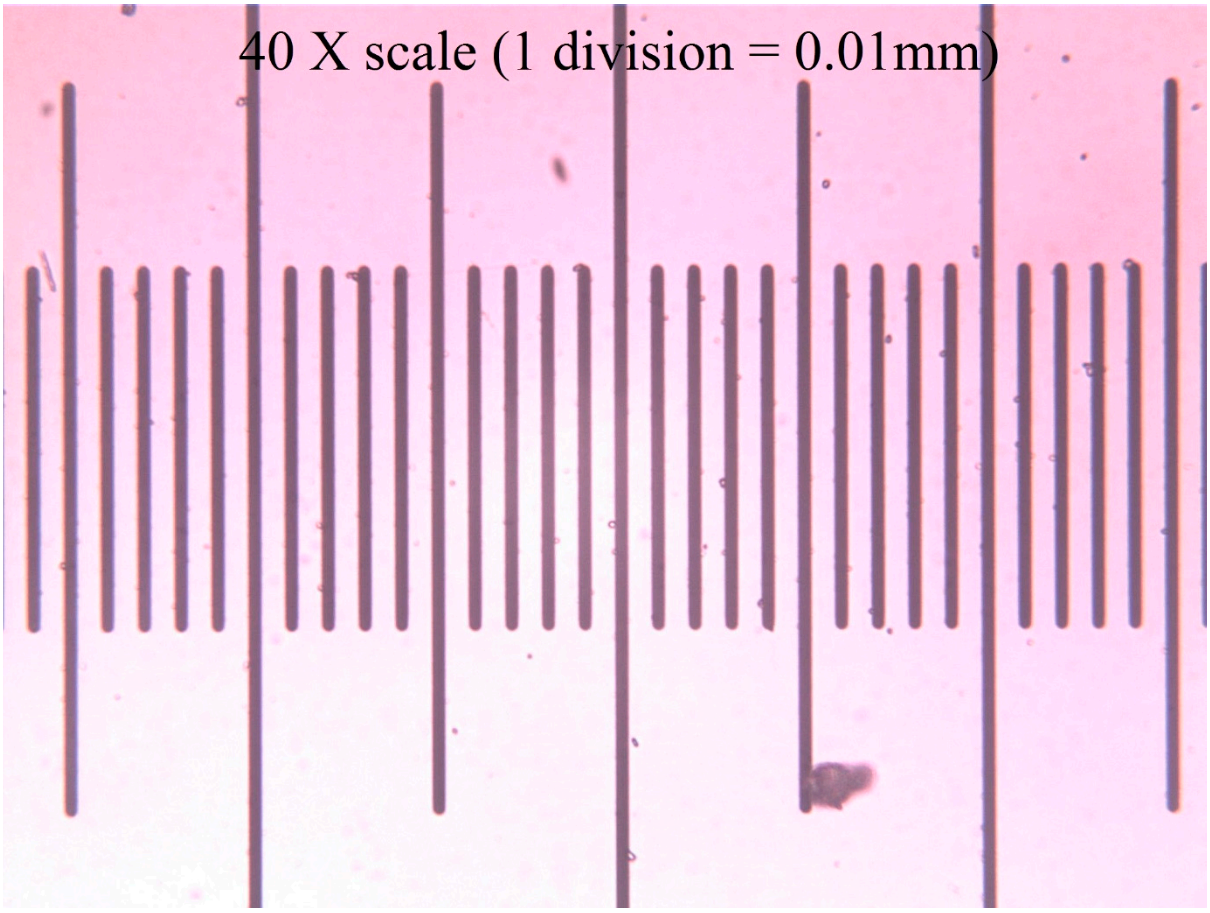

Supplemental figure: 40X scale for histological calculations
